# Reassessing the Casual Effects of Genetically Predicted Obesity on Pelvic Organ Prolapse: Letter to the Editor on “Lifestyle factors, metabolic factors and socioeconomic status for pelvic organ prolapse: a Mendelian randomization study”

**DOI:** 10.1186/s40001-023-01312-2

**Published:** 2023-09-27

**Authors:** Zhangsendi Chen, Weiguo Wang, Hongbo He

**Affiliations:** Department of Integrated Traditional Chinese and Western Medicine, West China Hospital, Sichuan University, Chengdu, Sichuan China

Dear Editor,

We had carefully read the Mendelian randomization (MR) study entitled “Lifestyle factors, metabolic factors and socioeconomic status for pelvic organ prolapse: a Mendelian randomization study” [1] with great interest. In this article, the authors focus on the effects of various metabolic traits on pelvic organ prolapse in women and their MR study provided genetic evidence for the causal role of waist-to-hip ratio (WHR) and waist-to-hip ratio adjusted for BMI (WHRadjBMI) in the risk of pelvic organ prolapse (POP) development. For phenotypes of obesity, the authors used sex combination instruments. Since pelvic organ prolapse is a female-specific disease, in theory, the female-specific instrumental variable effects should be obtained from the GWAS of exposures to avoid the problem of sex heterogeneity as well as the incorrect causal inference results. We thank the authors for their important work, and we would like to share our more nuanced causal conclusions regarding obesity and pelvic organ prolapse that the three phenotypes of obesity have a predictable genetic pathogenic role in POP.

Mendelian randomization was also used as the analysis method in our study. The Genetic instruments for obesity were stratified by sex but not combined, given the gender of the source of the outcome data. We obtained data on body mass index (BMI) (*N* = 806,834 including 434,794 women), WHR (*N* = 697,734 including 381,152 women), and WHRadjBMI (*N* = 694,649 including 379,501 women) from a large meta-analysis of genome-wide association studies (GWASs) [2]. For the dataset of POP, summary statistics from the R8 release of FinnGen biobank analysis including 15,197 cases and 100,663 controls were used to reduce the sample overlap rate (finn-b-N14_FEMGENPROL).

We set the genome-wide association significance threshold at *P* < 5.0 × 10^–8^ to meet the relevance assumption. Palindromic SNPs and SNPs in linkage disequilibrium (LD) structure (*R*^2^ < 0.001 within 10,000 kb) were excluded. Exposure and outcome data were harmonized to ensure SNP effects were on the same allele. Confounding factors related to phenotypes were removed using the "PhenoScanner" online database (www.phosanner.medschl.cam.ac). MR analysis was conducted using random-effects inverse-variance weighting (IVW), MR-Egger, the simulation extrapolation (SIMEX) correction and weighted median methods. Heterogeneity was assessed through MR-PRESSO, Cochran's Q test, and funnel plots to identify outlier SNPs. MR-Egger regression was conventionally employed assuming non-differential measurement error in the SNP–exposure association (NOME assumption). However, the IGX2 statistic is a more relevant measure for MR-Egger than the F-statistic. If regression dilution I^2^ is less than 90%, violating the NOME assumption, a SIMEX analysis is required [3].

To assess the independent association between genetic predisposition to the mentioned exposures and the risk of POP, while considering confounders such as educational attainment (EA) [4], smoking initiation, and drinks per week [5], we conducted multivariate Mendelian randomization (MVMR) analyses using a random-effects IVW model. All statistical analyses were performed in R (version 4.2.2) using the packages "TwoSampleMR" (version 0.5.6) and "simex".

Based on our analysis, the results indicate a significant association between genetically predicted obesity and an elevated likelihood of developing pelvic organ prolapse among women (Fig. 1). For the phenotypes of obesity, in the primary analyses using IVW, the genetically predicted higher BMI [odds ratio (OR): 1.112; 95% confidence interval (95% CI) 1.004, 1.231; *P* = 0.041], WHR (OR: 1.236; 95% CI 1.118, 1.366; *P* < 0.001) and WHRadjBMI (OR: 1.235; 95% CI 1.136, 1.343; *P* < 0.001) increase the risk of POP (Additional file 1: Table S1). The associations between the genetic predisposition to the three obesity phenotypes and the risk of pelvic organ prolapse persisted after we further corrected for the confounding effects of EA, smoking initiation, and drinks per week using multivariate MR, with ORs of POP (OR: 1.415; 95% CI 1.117, 1.713; *P* = 0.022) per 1-SD increase in BMI, 1.583 (95% CI 1.233, 1.934; *P* = 0.010) per 1-SD increase in WHR, and 1.967 (95% CI 1.491, 2.444; *P* = 0.005) per 1-SD increase in WHRadjBMI, respectively, using the IVW method (Fig. 2) (Additional file 2: Table S2). Genetic instruments associated with obesity are shown in (Additional file 3: Table S3-S5).

**Fig. 1:**
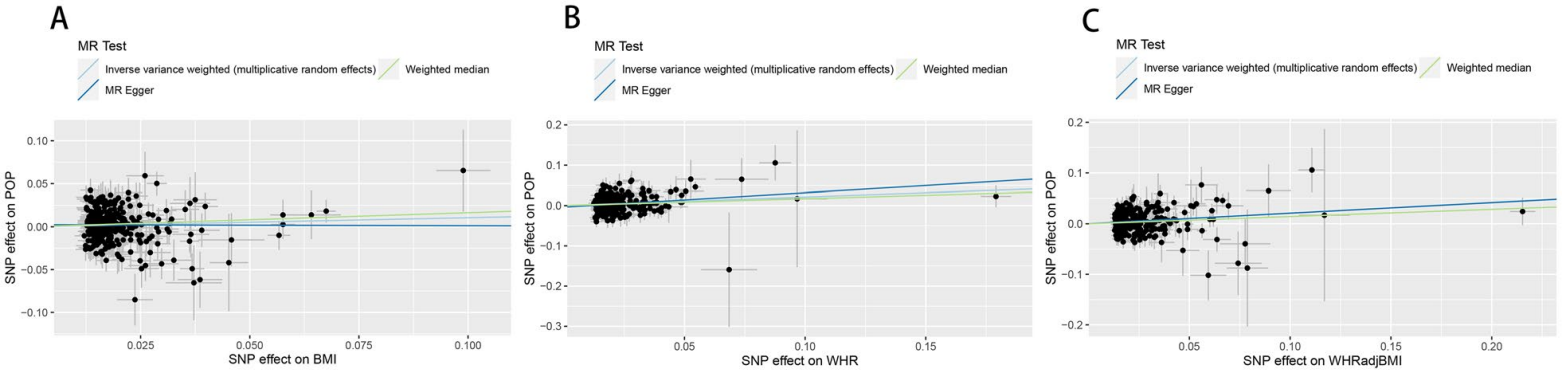
Scatter plots showing the genetic relationship between obesity and POP using inverse-variance weighted, MR-Egger, and weighted median, **A** BMI: body mass index; **B** WHR: waist-to-hip ratio; **C** WHRadjBMI: waist-to-hip ratio adjusted for BMI

**Fig. 2:**
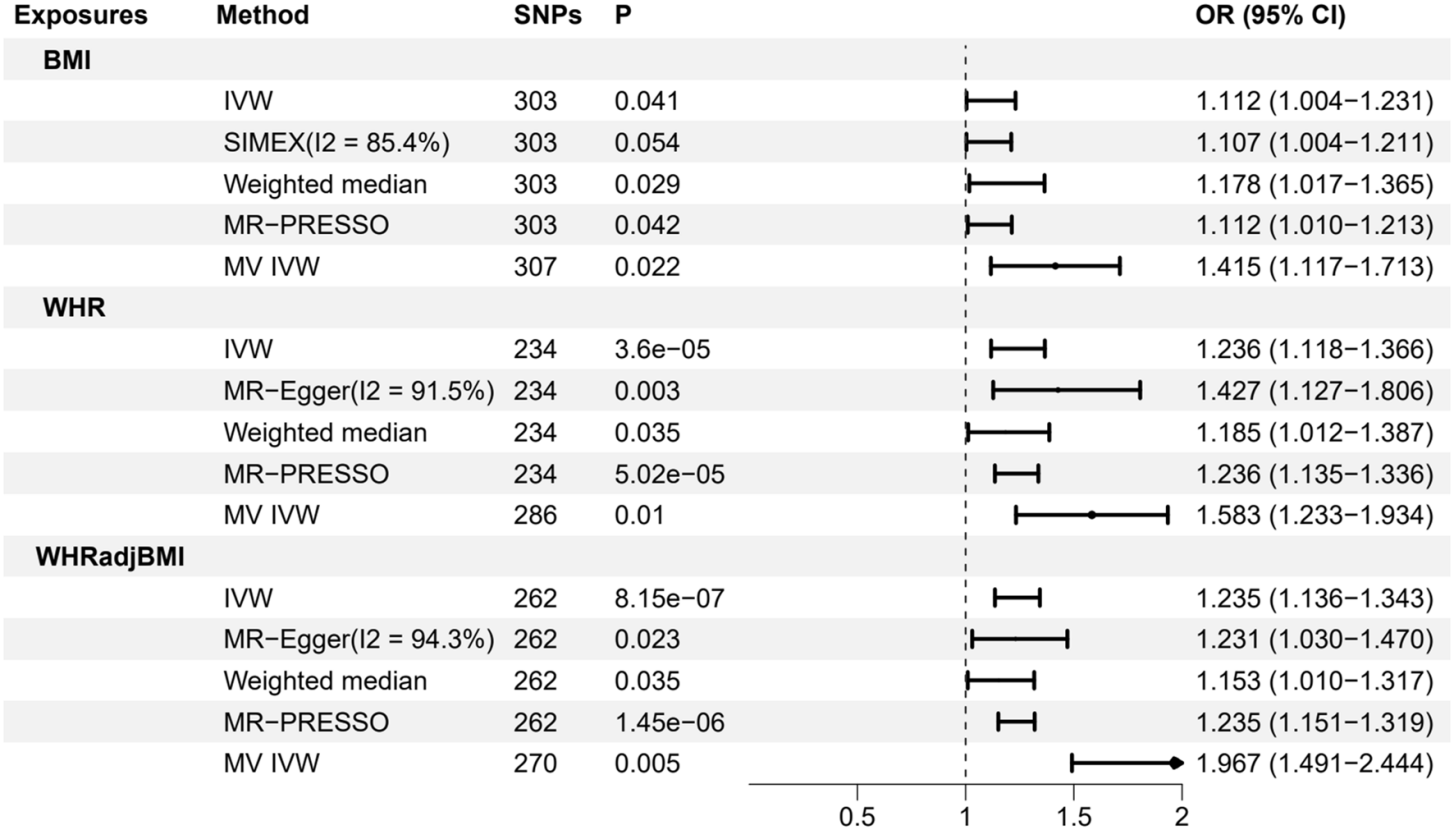
Forest plots for the results of UVMR and MVMR of obesity on the risk of POP, where MVMR controlled for EA, smoking initiation, and weekly alcohol consumption. SNP: single-nucleotide polymorphism; OR, odds ratio; CI, confidence interval; MR, Mendelian randomization; PRESSO, pleiotropy residual sum and outlier; MV IVW: multivariate Mendelian randomization (MVMR) analyses using a random-effects IVW model; BMI, body mass index; WHR: waist-to-hip ratio; WHRadjBMI: waist-to-hip ratio adjusted for BMI

Consistent with the authors, our analysis revealed significant instrumental heterogeneity (*P* < 0.05). Therefore, we used the random-effects IVW method with MR-PRESSO to remove outliers influenced by horizontal pleiotropy. MR-Egger intercept test showed no directional pleiotropy (*P* > 0.05). Since BMI's I^2^ statistic was < 90%, we employed SIMEX correction instead of MR-Egger to mitigate bias. Leave-one-out sensitivity analysis found no outlier SNPs affecting causal estimates, ensuring reliability.

Our study provides more detailed MR evidence supporting a significantly causal role of obesity in POP, which complements the conclusions of the study by Liu et al. and increases the feasibility of the results.

### Supplementary Information

**Additional file 1. Table S1**: Sensitivity analysis for Mendelian Randomization analysis.

**Additional file 2. Table S2**: Associations of obesity with POP adjusted for EA, smoking initiation, and drinks per day using multivariable Mendelian randomization.

**Additional file 3. Table S3**: Genetic information of SNPs associated with obesity for women in UVMR.

## Data Availability

The genetic association data for obesity is available at https://zenodo.org/record/1251813#.XxgQ2J5KiUl. The GWASs for POP were provided by FinnGen consortium (https://risteys.finregistry.fi/endpoints/N14_FEMGENPROL). The summary statistics for the GWAS related to the exposures and outcome can be accessed from the IEU GWAS database (https://gwas.mrcieu.ac.uk/).
